# Nanotechnological Strategies to Promote Skeletal Muscle Regeneration in Aging

**DOI:** 10.3390/ijms27146167

**Published:** 2026-07-10

**Authors:** Flavia Carton, Manuela Malatesta

**Affiliations:** 1 Center for Medical Sciences (CISMed), University of Trento, 38122 Trento, Italy; 2 Department of Cellular, Computational and Integrative Biology (CIBIO), University of Trento, 38123 Trento, Italy; 3 Department of Neurosciences, Biomedicine and Movement Sciences, University of Verona, 37134 Verona, Italy; manuela.malatesta@univr.it

**Keywords:** skeletal muscle, aging, sarcopenia, muscle atrophy, regenerative medicine, nanoparticles, extracellular vesicles, exosomes, nanostructured hydrogels

## Abstract

During aging, skeletal muscle undergoes a decline in mass and strength. This condition, known as sarcopenia, involves many physiological and metabolic impairments, thus representing a healthcare, social, and economic burden. Various pharmacological and non-pharmacological approaches have been explored to counteract sarcopenia; however, no definite treatment has so far been found. The present narrative review summarizes nanotechnology-based strategies designed to promote muscle preservation and functional recovery in aging. Synthetic organic or inorganic nanoconstructs and natural extracellular vesicles have been used as nanocarriers for drug delivery, have been active as intrinsic therapeutic agents, have been employed to build biomimetic nanoscaffolds to sustain muscle regeneration, or have been combined to form hybrid nanosystems with multiple therapeutic functions. These nanotools demonstrated promising results in vitro and in animal models, being able to counteract major factors responsible for sarcopenia, such as oxidative stress, inflammation, mitochondrial dysfunction, increased proteolysis, and impaired stem cell function. However, nanotools have mostly been tested on biological models far from the physiologically aged human muscle. Moreover, limitations still remain to be solved to make these nanotools suitable for regenerative medicine; in particular, the systemic administration requires nanoconstruct functionalization for skeletal muscle targeting, and proper clearance should be ensured to avoid toxicity and immunogenicity related to long-term use.

## 1. Introduction

Skeletal muscle is made of a variety of cell types, the main components being multinucleated fibers. These are active in movement and are classified into several fiber types, which differ in metabolic and functional properties [1,2,3].

The muscle tissue also contains a plurality of mononucleated cells: the postnatal myogenic stem cells (so-called satellite cells), and other non-muscle cell populations, such as fibroblasts, mesenchymal fibro-adipogenic progenitors, and immune, endothelial, and glial cells (e.g., the Schwann cells) [4,5]. All are involved in skeletal muscle homeostasis, and may influence the dynamics of both muscle fibers and satellite cells.

A decline in skeletal muscle mass and a decrease in strength and endurance progressively occur in aging, even in physically active healthy subjects, starting at the age of 30–50 in humans, with a rate of muscle loss of 1% to 8% per year [6]. This state is known as sarcopenia [7] and involves a number of physiological and metabolic impairments, among which compromised motor function and performance, and a higher risk of falls and premature death [8].

**Figure 1:**
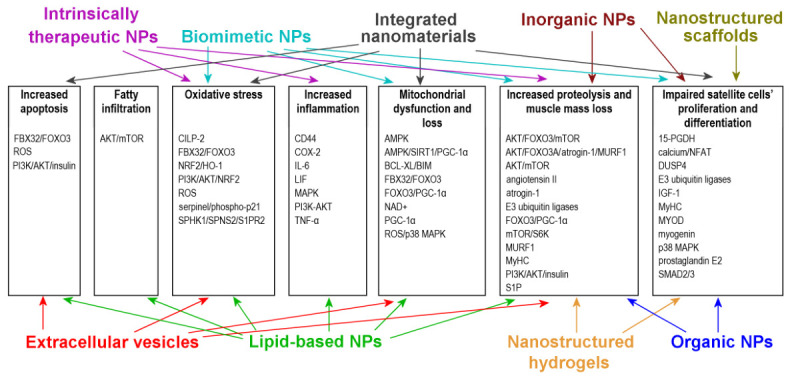
Schematic representation of the major mechanisms involved in sarcopenia, and of the main molecular factors/pathways targeted by the nanoconstructs described in the present review. Abbreviations: 15-PGDH, 15-hydroxyprostaglandin dehydrogenase; AKT, protein kinase B; AMPK, 5′ adenosine monophosphate-activated protein kinase; BCL-2, B-cell lymphoma 2; BCL-XL, B-cell lymphoma-extra large; BIM, BCL-2 interacting mediator of cell death; CILP-2, cartilage intermediate layer protein 2; COX-2, cyclooxygenase-2; DUSP4, dual specificity phosphatase 4; FBX32, F-box protein 32; FOXO3, forkhead box O3; HO-1, heme oxygenase-1; IGF-1, insulin-like growth factor-1; LIF, leukemia inhibitory factor; MAPK, mitogen-activated protein kinase; mTOR, mechanistic target of rapamycin; MuRF1, muscle RING finger protein 1; MyHC, myosin heavy chain; MYOD, myoblast determination protein 1; NAD+, nicotinamide adenine dinucleotide; NFAT, nuclear factor of activated T-cells; NPs, nanoparticles; NRF2, nuclear factor erythroid 2-related factor 2; PGC-1α, peroxisome proliferator-activated receptor gamma coactivator-1α; PI3K, phosphatidylinositol 3-kinase; ROS, reactive oxygen species; S1P, sphingosine-1-phosphate; S1PR2, sphingosine-1-phosphate receptor 2; S6K, ribosomal protein S6 kinase; SIRT1, sirtuin 1; SMAD2/3, small mother against decapentaplegic homolog 2 and 3; SPHK1, sphingosine kinase 1; SPNS2, spinster homolog 2; TNF-α, tumor necrosis factor α.

The age-dependent muscle wasting and weakness depend on multifactorial mechanisms that have not been fully elucidated. Some of the proposed hypotheses for sarcopenia occurrence point to muscle tissue factors (Figure 1), such as oxidative stress [9]; mitochondrial dysfunction and impaired mitochondrial dynamics and mitophagy [10,11,12]; increased proteolytic and autophagic activity, which alters protein homeostasis by promoting catabolic vs. anabolic pathways [13,14,15,16]; the loss of myonuclei, likely due to increased apoptosis [17,18,19], and an altered nuclear functionality with impaired RNA transcription and processing [20,21,22]; the decrease in the number of satellite cells and/or their reduced proliferation and differentiation potential [19,23,24,25]; the impaired function of cells regulating the extracellular microenvironment (especially the satellite cell niche), such as fibro-adipogenic progenitors [26,27,28], which play a key role in fatty infiltration and fibrosis that occur in aged muscles [29]. In addition, other factors have been found to contribute to sarcopenia, such as myofiber denervation [30,31], increased concentrations of inflammation mediators [32,33,34], a decrease in exercise tolerance and microvascular function [35,36], and impaired anabolic signaling due to an altered hormonal milieu [37,38].

Sarcopenia represents a serious healthcare and social problem, as a significant risk factor for weakness, invalidity, loss of independence, and even premature death in the elderly. In the attempt to design efficient therapeutic strategies to counteract the occurrence and progression of sarcopenia, various approaches have been explored that take into account the multiple factors responsible for muscle loss and wasting in aging.

Physical exercise is a widely used non-pharmacological approach to counteract sarcopenia since it has been proven to influence many age-related features [39,40], e.g., by promoting the expansion and functionality of satellite cells [41,42,43], improving mitochondrial activity [44,45], stimulating myonuclear activity [46,47], and increasing muscle mass [48,49]. Dietary intervention is another non-pharmacological approach that gives positive results in preventing and counteracting sarcopenia: appropriate food intake to avoid malnutrition and dehydration is, in fact, essential to preserve muscle mass and strength [50]. In particular, high-quality diets rich in proteins, creatine, vitamin D and B12, and fish-derived omega-3 fatty acids reduce the levels of inflammatory and cardiometabolic risk markers [51,52,53]; plant-derived bioactive substances (e.g., phytosterols, polyphenols, polyunsaturated fatty acids, vitamin E and C) confer anti-inflammatory and antioxidant properties, helping to preserve muscles [54], while amino acids such as leucine and glutamine stimulate muscle protein synthesis [55,56,57,58].

On the other hand, pharmacological approaches to counteract sarcopenia are limited due to the side effects in frail subjects often receiving polypharmacotherapy. However, immunomodulating drugs may contribute to combating sarcopenia by reducing the age-related inflammation that impairs muscle regeneration [59]. In addition, emerging pharmacological approaches include therapies based on stem cells [60,61], gene expression modulation to promote muscle regeneration [62,63], the administration of regulators of androgen receptors to promote an increase in muscle mass and strength [64,65], or of inhibitors of acetylcholinesterase to enhance the function of the neuromuscular junction [66,67]. However, most of these therapeutic agents are presently at the experimental or clinical trial stage, and have considerable side effects.

Over the last few years, nanotechnology has been widely used to explore innovative therapeutic treatments for various skeletal muscle diseases, leading to the development of effective and safe nanosystems for drug, gene, or nutrient delivery, and of nanostructured materials for muscle repair [68,69,70,71,72,73,74,75,76,77,78,79,80].

The present narrative review summarizes the nanotechnology-based strategies designed with the aim of limiting atrophy and promoting regeneration in aging muscle. Regenerative medicine aims at “replacing or regenerating human cells, tissue or organs, to restore or establish normal function” [81] and the articles reviewed in the following chapters demonstrate that nanomedical devices may contribute to the restoration of some age-related structural and functional muscle defects by carrying therapeutic agents to muscle cells, or by providing nanostructured biomimetic scaffolds.

## 2. Literature Search

For this narrative review, studies relevant to the topic were selected in PubMed and Scopus databases by using the keyword combinations (sarcopenia OR (muscle aging)) AND (nano* OR liposom*). The search period ranged from January 2000 to April 2026. Publications were first screened based on title and abstract content to discard those obviously dealing with subjects different from the review topic. The remaining articles were evaluated in full to ensure they dealt with nanoconstructs for sarcopenia or muscle aging. To be included, the publication had to involve aging/old human or animal (mammals) subjects; in the case of in vitro studies, they had to use skeletal muscle cells. Studies written in a language other than English, with incomplete/unclear methodologies, or missing outcome data were excluded. Finally, the literature search was refined by cross-referencing citations from the included studies in order to ensure comprehensive coverage of the topic.

## 3. Nanomaterials as Delivery Carriers

### 3.1. Organic Nanoparticles as Delivery Systems

**Figure 2:**
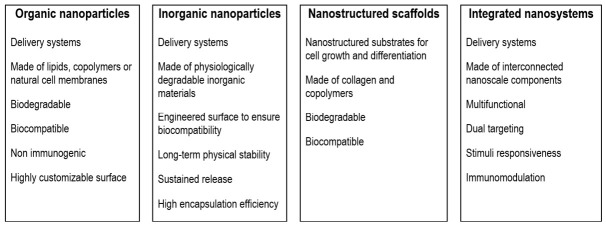
Schematic summary of the main features of the nanoconstruct classes treated in this review.

Organic nanoparticles (NPs) represent a versatile nanotechnological tool to counteract skeletal muscle decline and promote functional recovery in aging. These systems can deliver therapeutic agents, from small-molecule drugs to genetic material. The primary advantages of these organic systems include their excellent safety profile, their ability to protect the molecular cargo from enzymatic degradation, and a high degree of surface customizability for muscle-specific targeting (see Figure 2 for a summary of the main features of these nanoconstructs).

In particular, lipid-based NPs have been widely explored as delivery systems to counteract age-related muscle atrophy, as reported in the following studies (a summary can be found in Table 1).

Animal models of physiological aging represent the gold standard for studying primary sarcopenia. These models effectively recapitulate the complex causes of human muscle loss, such as genomic instability, chronic denervation, and metabolic decline.

Within this framework, Ishida et al. [82] used liposomes as a delivery vector for sphingomyelin, a cargo useful to counteract age-related muscle atrophy by enhancing the proliferation of myoblasts and stimulating muscle regenerative processes through sphingosine-1-phosphate (S1P) signaling. These liposomes were designed to target skeletal muscle, acting via the S1P signaling pathway. In a senescence-accelerated mouse prone 1 (SAMP1) model, oral administration of sphingomyelin-based liposomes significantly increased the weight of the quadriceps femoris muscle by approximately 1.5-fold compared to controls and extended swimming time until fatigue, suggesting that this system was able to improve senescence-associated muscle weakness and physical performance decline.

Another experimental approach utilizes secondary atrophy models. These models simulate specific clinical conditions or a biochemical stimulus (e.g., chronic glucocorticoid exposure) or mechanical unloading to induce muscle wasting.

In this context, in a rat model of glucocorticoid-induced muscle atrophy and osteopenia, Pal et al. [83] successfully employed a lipid-based self-nanoemulsifying drug delivery system to deliver an osteogenic butanolic fraction from *Cassia occidentalis* L. By oral administration, this formulation enhanced the bioavailability of key phytochemicals, which in turn protected rats from glucocorticoid-induced muscle atrophy and osteopenia. This protective effect was mediated by downregulating skeletal muscle atrogenes, specifically atrogin-1 and the muscle RING finger protein 1 (MuRF1). By suppressing these proteolytic markers, the treatment successfully preserved overall lean mass (maintaining it at 81.23%) and body weight (308.02 ± 8.47 g) in the treated group, effectively shielding the rats from muscle atrophy induced by methylprednisolone.

Another chemical induction approach mimicking certain aspects of aging through chronic oxidative stress and systemic inflammation is based on D-galactose. Huang et al. [84] employed D-galactose-induced aging mice to evaluate M12 muscle-homing peptide-modified liposomes as a targeted delivery system for epigallocatechin gallate to skeletal muscle. Following systemic administration via tail vein injection, these liposomes demonstrated higher muscle-targeting capabilities compared to non-modified liposomes. The treatment successfully modulated the mitogen-activated protein kinase (MAPK) and phosphatidylinositol 3-kinase (PI3K)- protein kinase B (AKT) signaling pathways, leading to reduction in pro-inflammatory cytokines (tumor necrosis factor α (TNF-α), interleukin-6 (IL-6)), a decrease in oxidative stress via the nuclear factor erythroid 2-related factor 2 (NRF2)/ heme oxygenase-1 (HO-1) axis, and restoration of mitochondrial function and energy metabolism in aging skeletal muscle.

Models involving acute damage or immunogenic stimuli (such as toxins, lipopolysaccharides, or structural trauma) represent another experimental approach used to investigate muscle recovery capacity and inflammatory vulnerability in the context of aging.

Within this framework, ultra-deformable liposomes were developed [85] as a high-efficiency delivery vector for perindopril erbumine. While traditionally indicated for hypertension, perindopril erbumine was explored for its ability to mitigate age-related muscle atrophy by inhibiting angiotensin II-mediated catabolism. Utilizing a mouse model of lipopolysaccharide-induced muscle atrophy, the authors demonstrated that topical perindopril erbumine-ultra-deformable liposomes administration significantly attenuated muscle loss and improved functional grip strength. At the molecular level, these improvements were characterized by the restored expression of myosin heavy chain (MyHC) and the concomitant downregulation of the E3 ubiquitin ligases, atrogin-1 and MuRF1, thus effectively reversing the proteolytic signaling associated with muscle wasting.

Similarly, to simulate muscle degeneration and regeneration defects, Santiago et al. [86] developed lipid NP-mediated delivery of Wnt Family Member 7A (*WNT7A*) mRNA, as a novel strategy for mitigating skeletal muscle degeneration in chronic injuries, degenerative myopathies, and aging. By acting on fibro-adipogenic progenitors and myofibers, the treatment activates the AKT/ mechanistic target of rapamycin (mTOR) pathway to promote tissue recovery. Lipid NPs were delivered to in vitro and in vivo models of glycerol-induced muscle injury. In mice, where lipid NPs were administered via repeated intramuscular injection, the therapy successfully increased myofiber size and significantly reduced fatty infiltration.

Finally, several studies have utilized an in vitro model of hydrogen peroxide-induced oxidative stress in muscle cells to simulate the oxidative microenvironment of aged muscle.

Najm et al. [87] developed self-assembled dipalmitoylphosphatidylcholine (DPPC) lipid vesicles as vectors to deliver three distinct drugs involved in promoting the health of muscle tissue: beta-hydroxy-beta-methylbutyrate (HMB), nicotinamide mononucleotide (NMN), or L-leucine. These formulations targeted the mitochondrial dysfunction and oxidative stress pathways in an in vitro C2C12 myotube model of aged muscle, induced by hydrogen peroxide treatment to simulate oxidative stress and muscular atrophy, by providing a controlled release of the loaded molecules. This synergistic approach collectively reduced the levels of reactive oxygen species (ROS) while restoring nicotinamide adenine dinucleotide (NAD+), a key mediator of mitochondrial quality and redox metabolism. This targeted delivery demonstrated that the drug-loaded vesicles effectively mitigated oxidative damage and preserved mitochondrial membrane potential, with HMB-loaded vesicles showing the most pronounced protective effect. In another study, Najm and collaborators [88] developed a hybrid liposomal platform composed of 1,2-dimyristoyl-sn-glycero-3-phosphocholine (DMPC) and 1,2-dioleoyl-sn-glycero-3-phosphocholine (DOPC) phospholipids for the dual-delivery of caffeine and hyaluronic acid methacrylate (HAMA) to treat some sarcopenia-inducing factors. These agents are useful because they address the multifactorial nature of the disease: caffeine acts as a metabolic and antioxidant agent that stimulates mitochondrial biogenesis and mimics exercise-induced signaling (e.g., IL-6 production), while HAMA focuses on maintaining the extracellular matrix integrity and supporting the muscle stem cell niche. Utilizing the in vitro model of hydrogen peroxide-treated C2C12 myotubes, the authors demonstrated that this co-delivery system targets the 5′ adenosine monophosphate-activated protein kinase (AMPK) and peroxisome proliferator-activated receptor gamma coactivator-1α (PGC-1α) signaling pathways to promote mitochondrial biogenesis. Simultaneously, the efficacy of HAMA was evaluated through its ability to improve cell viability, reduce apoptosis, and moderate ROS levels. Although this experimental model does not fully mimic the complex structure of the muscle tissue, the results highlight the role of HAMA in modulating the cellular microenvironment via the CD44 receptor-mediated signaling: this elicits anti-inflammatory effects and may be envisaged to protect resident muscle cells from oxidative damage, thereby supporting the necessary conditions to re-establish the muscle stem cell niche. This synergistic strategy effectively mitigated oxidative stress in vitro by significantly attenuating ROS levels and by preserving mitochondrial function by stabilizing the membrane potential. Furthermore, the formulation exerted potent anti-apoptotic effects, downregulating programmed cell death pathways in damaged C2C12 myotubes.

DMPC-based liposomes were also employed as a synthetic lipid vector to co-encapsulate NMN and Matrigel: this dual-delivery system was designed to restore NAD+ levels and enhance satellite cell activity by acting on mitochondrial respiration and muscle regeneration pathways [89]. When tested in an in vitro experimental model of hydrogen peroxide-induced atrophy of C2C12 myotubes, this formulation successfully enhanced the activity of satellite cells, reduced oxidative stress, and restored mitochondrial membrane potential.

Additionally, the in vitro study by Ishida et al. [82] demonstrated that sphingomyelin-based liposomes significantly increased the transport of sphingomyelin across Caco-2 cell layers (15.4% transport efficiency in Caco-2 cells), indicating improved intestinal permeability, and markedly stimulated the proliferation of C2C12 myoblasts (1.2-fold increase), confirming specific bioactivity for muscle cell growth.

The in vitro validation by Huang et al. [84] in C2C12 cells mirrored the in vivo findings, confirming that M12-modified liposomes attenuate oxidative stress and modulate MAPK and PI3K/AKT pathways directly at the cellular level.

Finally, the in vitro characterization by Santiago et al. [86] demonstrated that *WNT7A* mRNA-loaded lipid NPs effectively reduced adipogenesis in fibro-adipogenic progenitors while promoting myofiber hypertrophy in cultured cells.

Recent nanomedical advancements have also been focusing on lipid-based nanocarriers to enhance the bioavailability and therapeutic efficacy of anti-inflammatory compounds on skeletal muscle, utilizing distinct in vitro setups to directly evaluate muscle inflammation targeting. For instance, Mückter et al. [90] explored the potential of the natural hydrophobic sesquiterpene alcohol, farnesol, to mitigate inflammation in primary human skeletal myoblasts treated with specific unsaturated free fatty acids. The study compared the delivery capability of different liposomal architectures, specifically small unilamellar vesicles and multilamellar lipid-based vesicles: notably, farnesol-loaded small unilamellar vesicles were highly effective, drastically reducing the free fatty acid-induced expression of IL-6 and leukemia inhibitory factor. In contrast, free farnesol alone had no significant effect on human skeletal myoblasts, due to its poor bioavailability. Similarly, Maretti et al. [91] encapsulated the anti-inflammatory compound, palmitoylethanolamide (PEA), within solid lipid NPs made of a binary lipid matrix of stearic acid and cholesteryl stearate; the research focused on the potential of the PEA-solid lipid NPs to target inflammatory processes and the cyclooxygenase-2 (COX-2) pathways in C2C12 myoblasts. These NPs were successfully internalized by the myoblasts without inducing cytotoxicity, suggesting the potential of these nanocarriers as an innovative and side-effect-free tool to set up nanotechnological strategies against age-related muscle atrophy.

Ethosomes and transethosomes were also investigated as lipid-based vectors for transdermal delivery of cholecalciferol (vitamin D3), an agent known to exert anti-inflammatory properties [92] and to limit loss of muscle mass and function in the elderly [93,94]. Both ethosomes and transethosomes were found to efficiently and safely enter cultured keratinocytes, fibroblasts, and myoblasts [95], and proved to cross the epidermal barrier in explanted human skin [96]. It is worth noting that these nanocarriers showed different intracellular fate and skin penetration and persistence, which makes them versatile tools for the sustained or fast release of transdermally delivered cholecalciferol.

**Table 1:** Lipid-based nanoparticles developed to counteract age-related muscle atrophy. The table summarizes the nanoparticle features, the experimental models where they were tested, the molecular factors or pathways involved in their action, and the main outcomes obtained.

Nanovector/Targeting Strategy	Cargo	Experimental Model	Factors/Pathways	Main Outcomes	Ref.
Sphingomyelin- and cholesterol-based liposomes	Sphingomyelin	In vitro: C2C12 myoblasts, human intestinal epithelial Caco-2 cells In vivo: SAMP1 mouse (10 to 20 weeks old); physiological aging model. Route: oral administration	IGF-1, S1P, sphingomyelin, plasma glucose, triglycerides, non-esterified fatty acids	In vitro: increased myoblast proliferation and intestinal penetration In vivo: increased quadriceps femoris muscle weight, swimming time extension	[82]
Lipid-based self-nanoemulsifying drug delivery system	*Cassia occidentalis*	In vivo: male Sprague-Dawley rats; glucocorticoid-induced muscle atrophy and osteopenia Route: oral administration	atrogin-1, DMP-1, GILZ, IL-1β; MEPE, MuRF1; OPG, RANKL, *miR-29a, miR-17*, *miR-20a*	In vivo: preserved body mass, prevention of gastrocnemius muscle fiber atrophy and muscle wasting, upregulation of proteolytic markers	[83]
M12 muscle-homing peptide-modified liposomes	Epigallocatechin gallate	In vitro: C2C12 myoblasts/myotubes In vivo: C57BL/6 mouse (8 weeks old); D-galactose-induced aging model Route: intravenous administration	GDF8, GSH, IL-6, MAPK, MDA, MyHC I/II, MYOD, myogenin, NRF2/HO-1, PI3K/AKT, TNF-α, SOD, ATP, Na^+^-K^+^-ATPase, mitochondrial respiratory chain complexes I and IV;	In vitro: downregulation of pro-inflammatory markers, reduced oxidative stress, increased antioxidant enzymes, restoration of mitochondrial membrane potential and ATP production. In vivo: downregulation of pro-inflammatory cytokines; reduced oxidative stress, restoration of mitochondrial function and energy metabolism	[84]
Ultradeformable liposomes	Perindopril erbumine	In vivo: C57BL/6 mouse (8 weeks old); lipopolysaccharide-induced secondary atrophy Route: topical administration Ex vivo: skin obtained from abdominal area of healthy male Sprague Dawley rats	atrogin-1, MyHC, MuRF1	In vivo and ex vivo: skin permeation, restoration of grip strength, increased muscle weight and myofiber cross-sectional area	[85]
Lipid nanoparticle	*WNT7A* mRNA	In vitro: murine fibro-adipogenic progenitors, C2C12 myoblasts/myotubes In vivo: C57BL/6J mouse (12–14 weeks old); glycerol-induced muscle injury Route: intramuscular administration	AKT/mTOR, laminin, perilipin-1	In vitro: anti-adipogenic and pro-hypertrophic effects In vivo: increased myofiber size, reduced fatty acid infiltration	[86]
Self-assembled DPPC lipid vesicles	HMB, NMN, L-leucine	In vitro: C2C12 myoblats/myotubes	NAD+, mitochondrial membrane potential, ROS	In vitro: reduced ROS, NAD+ replenishment, maintenance of mitochondrial membrane potential	[87]
Hybrid liposomal platform composed of DMPC and DOPC phospholipids	Caffeine and HAMA	In vitro: C2C12 myotubes	AMPK/PGC-1α, ROS, mitochondrial membrane potential	In vitro: restoration of cell viability, decreased ROS level, maintenance of mitochondrial membrane potential and stimulated mitochondrial biogenesis, prevention of apoptosis	[88]
DMPC-based liposomes	NMN and Matrigel	In vitro: C2C12 myotubes	NAD+, ROS, mitochondrial membrane potential	In vitro: reduced oxidative stress, NAD+ replenishment, restored mitochondrial membrane integrity	[89]
Small unilamellar vesicles and multilamellar lipid-based vesicles	Farnesol	In vitro: Primary human skeletal myoblasts	CXCL8, CXCL12, IL-6, LIF	In vitro: anti-inflammatory action	[90]
Solid lipid NPs	PEA	In vitro: C2C12 myoblasts	COX-2, IL-6, TNF-α	In vitro: high myoblast internalization and excellent cytocompatibility of NPs	[91]
Ethosomes and transethosomes	Cholecalciferol	In vitro: C2C12 myoblasts/myotubes, keratinocytes (HaCaT cells), fibroblasts Ex vivo: human skin explants from abdominal plastic surgery	/	In vitro and ex vivo: high intracellular delivery, excellent cytocompatibility and deep transdermal permeation of the nanocarriers	[95,96]

Abbreviations: AKT, protein kinase B; AMPK, 5′ adenosine monophosphate-activated protein kinase; ATP, adenosine triphosphate; COX-2, cyclooxygenase-2; CXCL8 or 12, C-X-C motif chemokine ligand 8 or 12; DMP-1, dentin matrix protein 1; DMPC, 1,2-dimyristoyl-sn-glycero-3-phosphocholine; DOPC, 1,2-dioleoyl-sn-glycero-3-phosphocholine; DPPC, dipalmitoylphosphatidylcholine; GDF8, growth differentiation factor 8; GILZ, glucocorticoid-induced leucine zipper; GSH, glutathione; HAMA, hyaluronic acid methacrylate; HMB, beta-hydroxy-beta-methylbutyrate; HO-1, heme oxygenase-1; IGF-1, insulin-like growth factor-1; IL, interleukin; LIF, leukemia inhibitory factor; MAPK, mitogen-activated protein kinase; MDA, malondialdehyde; MEPE, matrix extracellular phosphoglycoprotein; mTOR, mechanistic target of rapamycin; MuRF1, muscle RING finger protein-1; MyHC I/II, myosin heavy chain I/II; MYOD, myoblast determination protein 1; NAD+, nicotinamide adenine dinucleotide; NMN, nicotinamide mononucleotide; NPs, nanoparticles; NRF2, nuclear factor erythroid 2-related factor 2; OPG, osteoprotegerin; PEA, palmitoylethanolamide; PGC-1α, peroxisome proliferator-activated receptor gamma coactivator-1α; PI3K, phosphatidylinositol 3-kinase; RANKL, receptor activator of nuclear factor kappa-Β ligand; ROS, reactive oxygen species; S1P, sphingosine-1-phosphate; SOD, superoxide dismutase; TNF-α, tumor necrosis factor α; *WNT7A*, Wnt family member 7A.

In the field of organic nanotechnologies, extracellular vesicles (EVs), including exosomes, represent promising non-invasive and naturally biocompatible tools for counteracting skeletal muscle decline and supporting tissue homeostasis (summary in Table 2).

By using physiological aging models, Qin et al. [97] employed adipose-derived EVs as therapeutic vectors for *miR-146a-5p* to regulate mitochondrial autophagy and delay aging in skeletal muscle. By targeting F-box protein 32 (FBX32), a crucial E3 ubiquitin ligase, these EVs modulated the FBX32/ forkhead box O3 (FOXO3) signaling axis. Following intramuscular injection into in vivo aged mice, the delivery of *miR-146a-5p* successfully attenuated mitochondrial autophagy, reduced ROS levels, and inhibited apoptosis, consequently delaying skeletal muscle aging.

Secondary atrophy models, such as those induced by mechanical unloading, denervation, or catabolic drugs like glucocorticoids, allow researchers to evaluate interventions against specific, accelerated muscle-wasting pathways. For instance, Chen et al. [98] utilized engineered EVs derived from C2C12 myoblasts as delivery vectors to transport CRISPR-associated protein 9 (CRISPR/Cas9)-based gene-editing system to target *miR-29b*. EVs-Cas9-29b were able to effectively reverse the atrophic features of mouse muscles in vivo after immobilization and denervation. In particular, following intramuscular injection into the gastrocnemius muscle for 5 weeks, EVs-Cas9-29b significantly increased gastrocnemius muscle weight by approximately 22.5% as well as myofiber cross-sectional area. These improvements were achieved through the activation of the AKT/FOXO3/mTOR signaling pathway.

Qi and colleagues [99] developed plant-derived EVs isolated from Chinese leek to deliver bioactive components to skeletal muscle. It has been demonstrated that Chinese leeks contain molecules, such as selenium-associated proteins and flavonoids, exhibiting potent anti-inflammatory and antioxidant properties. The Chinese leek EVs were validated in vivo in a dexamethasone-induced sarcopenic mouse model. The mice received Chinese leek EVs via oral administration, demonstrating the stability and systemic efficacy of these plant-derived EVs. The results showed that Chinese leek EVs ameliorate muscle wasting by activating the AMPK/sirtuin 1 (SIRT1)/PGC-1α axis to enhance mitochondrial biogenesis and mitophagy, while simultaneously inhibiting the AKT/FOXO3/atrogin-1/MuRF1 proteolytic signaling to maintain myosin homeostasis.

Several EV-based platforms were screened using in vitro C2C12 myoblasts or myotubes. For instance, Di Felice et al. [100] investigated muscle wasting and cachexia using C2C12 myoblasts genetically engineered to secrete nanovesicles enriched with heat shock protein 60 (HSP60). When applied to naïve muscle cells, these HSP60-loaded vesicles significantly upregulated the PGC-1α isoform 1 pathway, a key regulator of mitochondrial biogenesis involved in the prevention of muscle atrophy. This intervention effectively recapitulated the molecular benefits of physical exercise at the cellular level.

Qin et al. [97] complemented their aged-mouse data with in vitro assays, demonstrating that the delivery of *miR-146a-5p* via adipose-derived EVs into C2C12 cells directly modulated the FBX32/FOXO3 signaling axis, reducing cellular oxidative stress and apoptotic signaling. Chen et al. [98] validated their CRISPR/Cas9-loaded EVs in vitro using C2C12 myotubes treated with dexamethasone, angiotensin II, or TNF-α. Irrespective of the atrophic stimulus, the EV treatment preserved myotube morphology and suppressed the key ubiquitin-proteasome markers MuRF1 and atrogin-1. Qi and colleagues [99] confirmed in vitro the molecular mechanism of their plant-derived Chinese leek EVs using a model of dexamethasone-induced C2C12 myotube atrophy. The in vitro data mirrored the in vivo findings, confirming that the down-regulation of atrogin-1 and MuRF1 and the activation of mitochondrial quality control pathways occur within the targeted muscle cells.

Besides lipid-based NPs and EVs, alternative organic vectors have been engineered as nanotechnological tools to enhance muscle repair in aging (summary in Table 3).

In this regard, physiological aging and accelerated senescence models allow for the evaluation of therapeutics against the complex background of chronic, multi-systemic decline, such as the natural loss of satellite cell competence and gradual metabolic exhaustion. Polyethylene glycol-distearoylphosphatidylethanolamine (PEG-DSPE)-based NPs were functionalized to deliver the small-molecule inhibitor SW033291, which specifically targets the 15-hydroxyprostalandin dehydrogenase (15-PGDH) enzyme, thereby increasing muscle levels of prostaglandin E2, whose decline in aging muscle contributes to satellite cell dysfunction and reduced regenerative capacity. When evaluated in senescence accelerated mice-prone 8 (SAMP8, a mouse strain displaying a phenotype of accelerated aging), this targeted approach significantly enhanced muscle mass and physical function compared to non-targeted treatments [101].

The secondary atrophy model was employed by Xie et al. [102], who designed engineered biomimetic NPs coated with skeletal muscle cell membranes and conjugated with the muscle-homing peptide M12 to ensure high-precision targeting for curcumin delivery. In vivo validation using an accelerated aging model based on daily injections of D-galactose proved that systemic intravenous administration of these biomimetic NPs every other day for six weeks significantly improved motor ability and muscle metabolism. At the molecular level, this targeted delivery system successfully regulated the sphingosine kinase 1(SPHK1)/spinster homolog 2 (SPNS2)/sphingosine-1-phosphate receptor 2 (S1PR2) axis, reducing both chronic inflammation and α-synuclein expression. In another study, Huan et al. [103] evaluated the therapeutic efficacy of an oil-in-water nanoemulsion to mitigate skeletal muscle atrophy. The system was based on whey protein isolate for the encapsulation and oral delivery of astaxanthin and was tested in vivo utilizing a dexamethasone-induced mouse model of muscle wasting. Daily oral administration of nanoemulsion effectively mitigated skeletal muscle atrophy. This protective outcome was mediated through the FOXO3/PGC-1α signaling pathway, which concurrently enhanced muscle protein synthesis, reduced catabolic degradation by inhibiting the ubiquitin ligases, MuRF1 and atrogin-1, and suppressed excessive mitochondrial autophagy.

In vitro platforms utilizing immortalized cell lines or chemically induced aging surrogates provide an essential framework for verifying initial cellular uptake, biocompatibility, and targeted intracellular signaling cascades prior to systemic in vivo testing.

For instance, Maretti et al. [104] developed hybrid poly(lactic-co-glycolic acid) (PLGA)-lipid NPs to encapsulate PEA, an endogenous lipid mediator with anti-inflammatory activity. By integrating stearic acid and the surfactant Gelucire^®^ into the PLGA matrix, the authors optimized encapsulation efficiency and drug solubility. When screened in vitro on C2C12 myoblasts, these hybrid NPs demonstrated efficient cellular internalization and improved cell viability, indicating their potential for targeting inflammation-related pathways in aged skeletal muscle. Xie et al. [102] complemented animal data with an in vitro model of accelerated cellular senescence using D-galactose-treated C2C12 myoblasts. These biomimetic NPs directly rescued muscle cell dysfunction by significantly reducing cellular senescence markers (such as β-galactosidase) and restoring myogenic differentiation through upregulation of myoblast determination protein 1 (MYOD) and myogenin.

### 3.2. Inorganic Nanoparticles as Delivery Systems

While organic NPs offer significant advantages, their adoption can be hindered by poor long-term physical stability, ‘burst release’ kinetics, and low encapsulation efficiency. These limitations have catalyzed the development of inorganic alternatives (summary in Table 4).

In this context, by utilizing non-aged animal models where muscle wasting is triggered by specific systemic or local conditions, Yu et al. [105] developed diselenide-bridged mesoporous silica NPs to carry a dual cargo of selenium (as part of the NP framework) and leucine (loaded within the pores). This nanosystem was designed to maintain muscle homeostasis by enhancing the mTOR/ ribosomal protein S6 kinase (S6K) pathway for protein synthesis and inactivating the AKT/FOXO3/MuRF1 pathway to reduce protein degradation. Using dexamethasone-induced or denervation-induced mouse models of muscle atrophy, it was demonstrated that oral administration of these NPs effectively restored muscle mass and strength while maintaining a favorable safety profile compared to traditional supplements.

In another study, Xu et al. [106] developed lipoic acid-modified gold NPs (LA-Au NPs) to modulate the immune response in sarcopenic muscles, utilizing a glycerol-induced muscle injury mouse model. The results demonstrated that both local injection of LA-Au NPs and transplantation of nanoparticle-preconditioned macrophages (Mac@Au NPs) proved to be highly safe, showing no systemic toxicity. Both strategies significantly restored body weight, increased skeletal muscle mass, and enhanced functional strength and endurance. Ultimately, the macrophage-shuttle therapy with Mac@Au NPs demonstrated superior therapeutic efficacy by significantly boosting angiogenesis and accelerating functional tissue repair.

Cell culture models have also helped investigate how inorganic NPs may be suitable to counteract age-related muscle atrophy.

Specifically, Yu et al. [105] found that mesoporous silica NPs successfully reversed dexamethasone-induced atrophy in C2C12 myotubes. The system acted by scavenging intracellular ROS, protecting mitochondrial membrane potential, and inhibiting muscle protein degradation via AKT pathway activation and subsequent FOXO3/MuRF1 axis suppression. On the other hand, in vitro experiments by Xu et al. [106] using RAW 264.7 macrophages revealed that these mesoporous silica NPs were internalized in a dose- and size-dependent manner. Remarkably, 4 nm NPs significantly influenced macrophage polarization, shifting the balance from the pro-inflammatory M1 phenotype to the tissue-repairing M2 phenotype. This polarization was achieved through metabolic reprogramming, specifically by enhancing lysosomal autophagy and mitochondrial oxidative phosphorylation. These anti-inflammatory M2 macrophages further regulate C2C12 cells activity by promoting their proliferation and differentiation while inhibiting apoptosis under inflammatory conditions.

### 3.3. Nanoparticles as Intrinsic Therapeutic Agents

While traditional nanomedicine usually utilizes NPs as “shuttles” for drug delivery, an emerging paradigm focuses on the intrinsic therapeutic properties of the nanomaterials themselves. Within this framework, both organic and inorganic NPs have been developed as active agents that directly interact with the cellular and molecular machinery of the muscle microenvironment (summary in Table 5).

To model specific pathophysiological features of muscle wasting, several studies evaluated nanotherapeutic interventions using established in vivo platforms of secondary muscle atrophy and chemically induced injury.

For instance, Zhou et al. [107] developed exosome-like NPs by using Gouqi plants, rich in saccharides and lipids, to improve skeletal muscle health. In detail, these natural Gouqi-delivery nanovesicles were developed to counteract dexamethasone-induced muscle atrophy in C57BL/6J mice and C2C12 cells. The results showed that these nanovesicles effectively counteract muscle atrophy by increasing muscle fiber cross-sectional area and enhancing physical grip strength in mouse models. Mechanistically, these nanovesicles function as potent anti-aging agents by activating the AMPK/SIRT1/PGC-1α signaling pathway, with AMPK activation serving as the primary driver. This molecular shift stimulates oxidative phosphorylation and improves mitochondrial energy metabolism, ultimately promoting muscle renewal and highlighting the potential of these plant-derived vesicles to treat skeletal muscle aging.

Lien et al. [108], investigated the therapeutic potential of cerium oxide NPs in a chemically induced sarcopenia model mediated by 4-hydroperoxy cyclophosphamide injections in mice. Weekly administration significantly increased muscle strength, enlarged the cross-sectional area of individual myofibers, and reduced pro-inflammatory cytokine levels.

In another work, Seok et al. [109] developed a nanoscale colloidal dispersion to deliver Kyungohkgo, a traditional oriental medicine with potential therapeutic benefits for skeletal muscle but characterized by poor availability. The nano-sized particles of Kyungohkgo were successfully tested in both in vitro (L6 rat myotubes) and in vivo (rat) models of dexamethasone-induced muscle atrophy. The in vivo findings confirmed that the treatment significantly suppressed body weight loss, restoring it by approximately 11% compared to the dexamethasone-alone group. Furthermore, the formulation preserved muscle mass, significantly increasing the weights of the gastrocnemius and soleus muscles by 11.5% and 21.7%, respectively, while increasing the muscle fiber cross-sectional area by 32.7%. Finally, the treatment mitigated tissue fibrosis by drastically reducing collagen deposition and connective tissue proliferation in the muscle tissue by 57.1%.

To isolate the direct effects of the nano-formulations on skeletal muscle cells, several authors utilized in vitro platforms (specifically C2C12 or L6 cell lines) mimicking aspects of sarcopenia such as oxidative stress, senescence, or glucocorticoid-induced atrophy.

For instance, Zhou et al. [107] utilized C2C12 myotubes to investigate the molecular mechanisms of Gouqi-derived exosome-like NPs. The authors demonstrated that these plant-derived nanovesicles function as potent anti-aging agents in vitro by activating the AMPK/SIRT1/PGC-1α signaling pathway, which ultimately stimulates oxidative phosphorylation and improves mitochondrial energy metabolism. Lien et al. [108] employed C2C12 cells to test the self-regenerative and antioxidative properties of cerium oxide NPs. The treatment successfully induced the downregulation of the cartilage intermediate layer protein 2 (CILP2) and activated the serpinel/phospho-p21 signaling pathway to scavenge ROS and inhibit cellular senescence. Finally, Seok et al. [109] tested a nanoscale colloidal dispersion of Kyungohkgo in L6 myotubes subjected to dexamethasone-induced atrophy. The treatment in vitro significantly restored myotube diameter by downregulating the mRNA and protein expression of MuRF1, atrogin-1, and their upstream regulator FOXO3.

## 4. Nanomaterials as Structural Scaffolds

In cases of severe injury or age-related decay, the loss or damage of the muscle extracellular matrix framework prevents proper tissue repair and functional recovery [110]. With the aim of effectively promoting skeletal muscle regeneration, a specialized microenvironment that mimics the structure of the natural extracellular matrix is needed. To restore functional muscle tissue, aligned nanofibers and micropatterned constructs have been developed to provide appropriate nanostructured scaffolds (see Figure 2 for a summary of the main features of these nanoconstructs). These scaffolds may act as physical templates to guide the alignment of myogenic cells, promote myofiber maturation, and facilitate neurovascular integration by supporting the infiltration of new blood vessels and the reinnervation of maturing fibers (a summary can be found in Table 6).

Several studies employed acute or surgical insults in animals to mimic the pathophysiology of age-related muscle atrophy. For instance, Habing et al. [111] used surgical ablation models to simulate severe musculoskeletal trauma and evaluate the capacity of engineered scaffolds to bridge large structural defects and promote neurovascularization. In particular, an engineered muscle construct consisting of a nanofibrillar-aligned collagen scaffold (with an average porosity of 11.3 ± 1.03% and a high anisotropy value of 0.804 ± 0.032), laden with green fluorescent protein (GFP)+ primary mouse myogenic cells, was transplanted into a murine model of volumetric muscle loss obtained by surgical ablation. Once the scaffold ability to promote optimal myotube formation and nuclear fusion was successfully validated in vitro, the construct was administered via direct surgical implantation into the defect site following a 20–30% volumetric muscle loss injury of the tibialis anterior muscle of young (8-week-old) and aged (80-week-old) mice. It was found that young mice had a significant response to the dual treatment, showing a 2.45-fold increase in muscle force production (286 ± 46 mN, *p* < 0.001) compared to age-matched controls, alongside a significantly improved muscle mass ratio (0.70 ± 0.063, *p* < 0.05) and enhanced vascular density (*p* < 0.01). In contrast, aged mice exhibited a diminished regenerative response, maintaining a lower total daily running distance (2.6-fold lower than young mice throughout the study), and showed no significant functional improvements in muscle force (204 ± 44 mN) or muscle mass ratio (0.53 ± 0.023) compared to their respective controls (189 ± 69 mN), while displaying persistently elevated inflammatory markers. This article demonstrated that the use of aligned nanofibrillar collagen scaffolds provides the topographical cues necessary for myogenesis and neurovascularization in volumetric muscle loss models; moreover, the results highlight that this regenerative rehabilitation approach acts through age-specific pathways, suggesting that regenerative therapies must be tailored across the lifespan to address the age-related limitations and differential healing responses observed in musculoskeletal trauma.

Chemical myotoxic models are also utilized to induce synchronous muscle fiber degeneration, allowing researchers to study the distinct phases of muscle stem cell activation, proliferation, and differentiation in vivo. Utilizing this platform, recent studies have demonstrated that nanotopographical cues can precisely modulate the therapeutic efficacy of stem cell-derived EVs. Specifically, Wang et al. [112] established that the physical culture substrate significantly dictates the functional cargo of EVs derived from young muscle stem/progenitor mouse cells. To introduce directional contact guidance and structural anisotropy, the researchers engineered polydimethylsiloxane nanogratings measuring 500 ± 13 nm in line width, 648 ± 28 nm in spacing, and 631 ± 50 nm in height. Functionally, EVs released by young stem/progenitor cells cultured on flat substrates (fEVs) improved the proliferation of aged stem/progenitor cells, while those from stem/progenitor cells cultured on nanogratings (nEVs) stimulated myogenic differentiation. Exploiting these divergent effects, the researchers demonstrated that administering fEVs followed by nEVs significantly enhanced aged muscle regenerative processes in a mouse model of cardiotoxin-induced injury of the tibialis anterior muscle. fEVs or nEVs were administered in vivo via intramuscular injection into the injured muscle. This strategy underscores the importance of precisely timed, stage-specific interventions in managing tissue repair and suggests that physical environmental cues can be effectively utilized to ‘program’ the biological activity of cell-free therapies.

The specific anti-aging properties of novel nanomaterials functioning as scaffolds have also been studied in vitro by using immortalized cell lines or primary cell cultures, including multi-cellular 3D constructs. Cai et al. [113] investigated the potential of the growth differentiation factor 11 to promote myogenic differentiation using a 3D co-culture system of human primary myoblasts and adipose-derived mesenchymal stromal cells. To reproduce the natural muscle architecture, the researchers developed electrospun-aligned poly-ε-caprolactone (PCL)-collagen I-polyethylene oxide (PEO) nanofibers, as a biocompatible matrix for cell growth and differentiation, which featured a mean fiber diameter of 233 ± 116 nm, an elastic Young’s modulus of 21.3 ± 7.3 MPa, and a highly oriented topography where 95.45% of the fibers fell within ±9.2° of the main axis. The study focused on the myogenic differentiation pathway, specifically monitoring the intracellular downstream signaling cascade and key terminal myogenic markers. Growth differentiation factor 11 supplementation under serum-free conditions successfully promoted myogenic differentiation of primary myoblasts and adipose-derived mesenchymal stromal cells co-culture, while upregulating *MYHC II* gene expression. In detail, at an optimized low concentration of 25 ng/mL, which yielded a significantly higher myotube fusion index (MFI = 0.159) at day 7 compared to serum-free medium alone (MFI = 0.071, *p* = 0.015) or a high dose of 500 ng/mL (MFI = 0.034), a significant upregulation of the *MYCH2* gene was found by day 28 (*p* = 0.021).

In a related study, a multiscale scaffold was fabricated by photolithographically patterning non-adhesive PEG hydrogel micro-lines (about 40 µm high, 377 µm clear channels) onto sub-micron electrospun PCL mats, where 70% of the fibers (776 nm in diameter) were highly aligned to mimic native myofibrils [114]. Subsequent in situ functionalization with gold NPs (15.65 nm) quadrupled surface roughness (S_a_ = 4.89 µm), rendered the porous topography highly hydrophilic (contact angle of 38°), and increased the local microscale stiffness to 764.3 kPa, while absolute electrical conductivity remained below the electroconductive threshold. This construct was used to evaluate the alignment and myogenic differentiation of C2C12 myoblasts. In vitro results confirmed that the scaffolds were non-cytotoxic and promoted a significant increase in cell proliferation by day 5, with gold NP-functionalized variants exhibiting the highest growth rates. Confocal microscopy demonstrated that the synergy of nanofiber guidance and PEG micro-confinement achieved precise structural alignment, limiting myotube deviation to within 15° of the intended axis. Furthermore, reverse transcription polymerase chain reaction analysis revealed that, while basal myogenic markers (myogenin and desmin) remained high, the PCL-gold NP-functionalized-PEGylated scaffolds specifically enhanced terminal differentiation through the upregulation of insulin-like growth factor-1 (IGF-1) and the E3 ubiquitin ligase. This work presents a multiscale approach for controlling the hierarchical organization and maturation of skeletal muscle tissue, offering a versatile platform that can be further enhanced through electrical or mechanical stimulation.

## 5. Integrated Nanotechnologies as Delivery Strategies

Nanomaterials can be developed as highly efficient systems that can target specific metabolic and signaling pathways to restore cellular homeostasis and counteract age-related muscle wasting. Emerging nanomaterials are distinguished by a dual function in the muscle (see Figure 2 for a summary of the main features of these nanoconstructs): they may either act as vectors for transporting bioactive signals (such as EVs and growth factors) or as biomimetic scaffolds activating specific receptors (summary in Table 7).

Physiological aging models based on aged animals are essential for evaluating the translational efficacy of biomaterials and advanced drug delivery systems in counteracting the structural and functional decline of muscle mass typical of sarcopenia.

By using this model, Lee et al. [115] developed an injectable, thermosensitive polaxamer hydrogel as a biocompatible and thermosensitive vehicle for the co-delivery of a protein cargo consisting of cluster of differentiation 146 (CD146), IGF-1, and collagen I/III. This platform facilitated a multi-stage recovery process: CD146 bound to the vascular endothelial growth receptor-2 (VEGFR-2), stimulating the recruitment of neutrophils and macrophages to promote the efferocytosis of necrotic debris, while IGF-1 drove satellite cell differentiation and myogenesis, and collagen I/III allowed the scaffold to evade host immune responses. The therapeutic potential of this synergistic platform was validated in vivo using aged (24-month-old) mice to confirm the age-related decline of CD146, and a lipopolysaccharide-induced muscle injury mouse model for therapeutic evaluation. In the latter model, a single subcutaneous injection of the hydrogel into the tibialis anterior muscle recruited M2 macrophages and successfully restored muscle mass by day 3, increasing the mean myofiber cross-sectional area.

In another work, exosomes derived from young, healthy tendon stem cells (h-TSC-Exos) were used as a therapeutic cargo in an aged chronic rotator cuff tear rat model [116]. These exosomes, which act as paracrine signaling vectors, were encapsulated in a releasable hyaluronic acid hydrogel and administered by local injection into the tendon-to-bone interface. This treatment targeted senescent tendon stem cells (s-TSCs) and macrophages inside the chronically torn tendon tissue. h-TSC-Exos were able to shift the macrophage polarization from the pro-inflammatory M1 phenotype to the anti-inflammatory and regenerative M2 phenotype, thus activating the bone morphogenetic protein 4 (BMP4)/small mother against decapentaplegic homolog 1, 5, and 9 (SMAD1/5/9) signaling pathway. This successfully attenuated s-TSC senescence (as marked by the reduced expression of p16 and p21) and rejuvenated multipotency for tenogenic, chondrogenic, and osteogenic differentiation needed for functional healing.

To address age-related muscle atrophy, Liu et al. [117] developed an innovative muscle-targeted nanocomposite vector by grafting the mitochondrial peptide 12S rRNA type-c (MOTS-c) onto PEGylated antioxidant black phosphorus nanosheets. This nanocomposite was evaluated in vitro in a D-galactose-induced senescence model in C2C12 myotubes and in vivo in aged mice. In both cellular and aged murine models, nanocomposite treatment significantly alleviated muscle cell atrophy and muscle dysfunction while concurrently normalizing mitochondrial function. Furthermore, the nanocomposite acted as a powerful antioxidant system, safely maintaining a hemolysis rate below 5%, drastically lowering mitochondrial ROS, and exerting a targeted immunomodulatory effect by selectively reducing the systemic levels of the key pro-inflammatory cytokines TNF-α and IL-6 in old mice. RNA- sequencing analysis revealed that the nanocomposite achieves these therapeutic effects by activating the PI3K/AKT/NRF2 pathway, which enhances cellular antioxidant defenses, and suppressing the ROS/p38 MAPK pathway, helping to maintain mitochondrial integrity and function.

Another widely used approach involves secondary atrophy models, which induce muscle loss through specific external stimuli unrelated to natural aging. A prime example is glucocorticoid administration (e.g., dexamethasone), which allows researchers to investigate therapeutic targets within precise catabolic pathways.

In this regard, an ultrashort peptide-based supramolecular hydrogel was developed by Shang et al. [118] to mimic IGF-1; this biomimetic nanomaterial acts through its own self-assembled nanofiber structure and targets the IGF-1 receptor, increasing the phosphorylation of AKT by activating the PI3K/AKT/insulin signaling pathway, which is critical for muscle growth and maintenance. Tested in vivo on a mouse model of dexamethasone-induced sarcopenia, the hydrogel effectively attenuated muscle tissue fibrosis and increased muscle mass by 5.01 g over 28 days of treatment.

Recently, Zhang et al. [119] developed a multifunctional myogenic nanomaterial for the treatment of systemic sarcopenia and orthopedic perioperative treatment. In detail, a two-dimensional layered double hydroxide vector was used to deliver urolithin A and metal ions (Mg, Al, Co). In this system, Mg and urolithin A promote myocyte proliferation and mitochondrial homeostasis, while Co stimulates angiogenesis, and Al acts as an immune adjuvant to enhance macrophage-derived glutamine nourishment for satellite cells. In a systemic dexamethasone-induced rat model of sarcopenia, the nanosystem significantly enhanced muscle mass, increased overall body weight, and improved grip strength (boosting it from ~290 g in the untreated group up to ~350–370 g), while promoting angiogenesis and restoring the dynamic balance and overall health of mitochondria in muscle cells. These therapeutic outcomes were achieved through the activation of the AMPK/SIRT1/PGC-1α and B-cell lymphoma-extra large (BCL-xL)/BCL-2 interacting mediator of cell death (BIM) pathways, alongside a drastic downregulation of muscle atrophy and an upregulation of myogenesis. The authors concluded that administration of this nanosystem may provide long-term improvement of age-related muscle wasting and short-term significant reduction in orthopedic perioperative complications.

In a more recent study [120], energy-replenishing hydrogel microspheres composed of aldehyde-modified HAMA were developed as a vector to deliver NMN, which was encapsulated in liposomes and targeted to the mitochondria via a grafted SS-31 peptide. Using experimental models of sarcopenia, induced by dexamethasone in C2C12 myotubes and mice, the researchers demonstrated that the treatment activates the AMPK/SIRT1/PGC-1α signaling pathway. The results revealed that this targeted delivery system significantly restored mitochondrial membrane potential and increased ATP production, while successfully alleviating cellular senescence through the marked downregulation of the key aging markers p16, p53, and p21. Furthermore, the nanoconstruct effectively prevented muscle fiber atrophy, significantly increasing myofiber number, cross-sectional diameter, and overall cross-sectional area, and markedly improved motor function in vivo, as demonstrated by enhanced stride strength, improved gait patterns, and increased standing duration in the treated animals.

Kanazawa et al. [121] developed an injured muscle-targeted drug delivery system using thiol-rich human serum albumin NPs as biomimetic vectors to deliver the antioxidant drug, edaravone, and treat sarcopenia. To evaluate this technology in a complex biological environment, the researchers utilized a hindlimb unloading mouse model, which effectively simulates the progressive muscle loss occurring in sarcopenia and exhibits significantly elevated secreted protein acidic and rich in cysteine (SPARC) levels. In vivo experiments showed that the NPs accumulate in damaged hindlimb muscle, significantly restoring skeletal muscle mass and endurance while suppressing myopathic factors like myostatin.

To address the therapeutic challenges of osteosarcopenia, Che et al. [122] engineered a unique dual-targeting hybrid nanovesicle system by fusing induced pluripotent stem cells (iPSC)-derived mesenchymal stem cell extracellular vesicles (iMSC-EVs) with peptide-modified liposomes. This biomimetic vector utilizes two specific ligands—SDSSD (Ser-Asp-Ser-Ser-Asp) for bone targeting and ASSLNIA (Ala-Ser-Ser-Leu-Asn-Ile-Ala) for muscle targeting—to achieve precise delivery of a *miR-206-5p* cargo. The system exhibits a highly structured multifunctionality and immunomodulatory capability, designed to simultaneously rescue bone marrow mesenchymal stem cells (BMSCs) and muscle satellite cells from cellular senescence. The therapeutic efficacy was validated in a murine model of osteosarcopenia. The dual-peptide modification successfully redirected the hybrid nanovesicles toward musculoskeletal tissues. These results culminated in the effective reversal of osteosarcopenia, characterized by a significant alleviation of both bone and muscle loss, restoration of mitochondrial function, and modulation of inflammatory macrophage phenotypes.

Acute injury models, provoked by toxic agents, endotoxic insults (lipopolysaccharide), or extensive physical trauma, are fundamental for studying tissue engineering processes linked to inflammation, the clearance of necrotic debris, and the regeneration kinetics of myofibers.

In this regard, Lyu et al. [123] adopted an original approach to promote muscle recovery, setting up an injectable piezoelectric hydrogel as a wireless electrotherapy vector to deliver ultrasound-activated lead-free (K, Na) NbO_3_ NPs. The system was tested in a murine model of volumetric muscle loss with full-thickness tibialis anterior defects. The treatment accelerated muscle and neural regenerative processes, driven by a rapid intracellular Ca^2+^ influx that activated the downstream calcineurin/nuclear factor of activated T-cells cytoplasmic 1 (NFATC1) signaling pathway. Furthermore, the system effectively modulated the immune microenvironment by polarizing macrophages from a pro-inflammatory M1 to a pro-healing M2 phenotype. This microenvironment modulation significantly reduced fibrosis, ultimately restoring hindlimb grip strength to 92% of uninjured muscle capacity and significantly improving locomotive gait patterns within 4 weeks of treatment.

Finally, in vitro models, predominantly based on immortalized C2C12 myoblasts, allow researchers to isolate biochemical and mechanical variables, identifying the early molecular pathways and myogenic markers activated by biomaterials.

For instance, by using C2C12 cells, Lee et al. [115] confirmed the effectiveness of the synergistic cargo (CD146/IGF-1/collagen) in inducing the efferocytosis of neutrophils and macrophages and promoting the transition of satellite cells into mature myofibers. This was corroborated by the upregulation of critical myogenic regulatory factors, including MYOD, myogenin, and MyHC II.

In another study, Shang et al. [118] tested the biomimetic supramolecular (IGF-1 mimic) hydrogel on C2C12 cell cultures. In vitro, this supramolecular hydrogel promoted proliferation, reduced apoptosis, and enhanced myotube differentiation through the up-regulation of the myogenic markers, driving a 2.26-fold increase in MYOD expression and a 1.74-fold increase in myogenin.

Zang et al. [119], evaluated the efficacy of a novel two-dimensional layered double hydroxide nano-adjuvant using mouse macrophage cells (RAW 264.7), human umbilical vein endothelial cells (HUVEC), primary mouse muscle satellite cells, and C2C12 cells to achieve sustained improvement in systemic and orthopedic-related sarcopenia. Results demonstrated that the system effectively reversed dexamethasone-induced toxicity and cellular senescence. Specifically, it significantly upregulated myogenesis-related genes (*Ccnd1*, *Myogenin*, *Murf1*, *Myf5*, *Myh7*), enhanced endothelial tube formation and angiogenesis in HUVEC, and restored mitochondrial homeostasis by lowering ROS and boosting ATP synthesis. Furthermore, it triggered a beneficial metabolic crosstalk, stimulating macrophages to secrete glutamine to actively nourish and activate muscle satellite cells.

In in vitro studies using H_2_O_2_-injured C2C12 myocytes, A549 human lung adenocarcinoma, and HepG2 human hepatocellular carcinoma cells, Kanazawa et al. [121] demonstrated that edaravone-loaded human serum albumin NPs were internalized in a SPARC-dependent manner via the formation of disulfide bonds with human serum albumin thiol groups. Once internalized, the NPs effectively reduced intracellular ROS.

In vitro cellular assays on murine BMSCs and muscle satellite cells demonstrated the successful delivery of *miR-206-5p* via dual-targeting hybrid nanovesicles. These nanovesicles, engineered by Che et al. [122], were fabricated by fusing iMSC-EVs with peptide-modified liposomes. This biomimetic cargo downregulated dual specificity phosphatase 4 (DUSP4) and activated p38 MAPK phosphorylation, which are involved in promoting osteogenic and myogenic differentiation.

In the study of Lyu et al. [123], in vitro assays using C2C12 cells demonstrated that the piezoelectric hydrogel nanosystem stimulated the calcium/nuclear factor of activated T-cells (NFAT) signaling pathway. In detail, it triggered a rapid influx of intracellular calcium, facilitating the nuclear translocation of the transcription of NFATC1, required for the correct transformation of muscle progenitors into myotubes, thus promoting the formation of mature myotubes through upregulated myogenin and MyHC expression and increased myoblast fusion.

## 6. Conclusions

The reviewed articles clearly demonstrate the great vitality and inventiveness of the nanotechnological research aimed at developing innovative and efficient strategies to counteract age-related skeletal muscle atrophy.

Various synthetic organic or inorganic nanoconstructs and natural EVs have been used as nanocarriers for drug delivery, have been active as intrinsic therapeutic agents themselves, have been employed to build biomimetic nanoscaffolds to sustain muscle regeneration, or have been combined to form hybrid nanosystems with multiple therapeutic functions. All the tested nanotools demonstrated promising results, contributing to limiting some events responsible for sarcopenia, such as oxidative stress, inflammation, mitochondrial dysfunction, increased proteolysis, and impaired stem cell proliferation and differentiation.

However, the tested nanotools are highly heterogeneous, as they substantially differ in many characteristics such as cargo compatibility, biodistribution, targeting potential, release kinetics, immunogenicity, biodegradability, and administration route. At the current state of knowledge, the most promising candidates for therapeutic application in the near future seem to be organic nanoconstructs like lipid-based NPs and hydrogels, thanks to their well-studied biocompatibility, high versatility in delivery efficiency and targeting potential, and low immunogenicity, as well as because in recent years they have been accepted by major regulatory bodies. However, all the studies reported in this review are at an early stage of nanotool development; therefore, it is impossible to predict which formulation will become successful as the research progresses.

It is worth noting that these studies have a significant limitation because the nanotools have mostly been tested on biological models far from the real, physiologically aged human muscle. In fact, most of the research has been performed on rodent models of secondary atrophy or acute injury/regeneration, or even on cultured muscle cell lines. These experimental models are very useful in the early design of novel nanoformulations intended for skeletal muscle, but only partially mimic the complex sarcopenic condition in humans, and may even exhibit regeneration mechanisms and immune responses distinct from those observed in aged muscle, thus providing little or even no information on the real nanoconstruct therapeutic potential. Moreover, many studies lack quantitative evaluation of the reported positive results on the aging muscle models, making it difficult to estimate the therapeutic potential of the nanoconstructs. Therefore, many research steps are still necessary for these quite promising nanotools to be translated into regenerative medicine.

It is known that sarcopenia affects almost all skeletal muscles of the body (although not uniformly or simultaneously); therefore, systemic (oral or intravenous) administration would be preferable to local (intramuscular or topical) application. Consequently, nanotools should be set up to overcome the biological barriers or mechanisms (e.g., the capillary walls, the dense extracellular matrix network, adhesive trapping, enzymatic degradation and opsonization in the blood, and kidney filtration) that physiologically protect the human body and could prevent them from reaching the skeletal muscle cells. Nanoconstructs should also be appropriately functionalized to ensure specific—or at least preferential—muscle targeting, as already investigated for some NPs [124,125,126].

Biocompatibility, biodegradability, and safety are crucial features for any nanoconstruct for therapeutic use, but these characteristics are especially important when long-term use with repeated dosing must be envisaged to treat elderly patients, characterized by chronic, multisystemic diseases and polypharmacy. Thus, a proper clearance of the systemically administered nanoconstructs must be ensured to avoid toxicity due to long-term accumulation in the liver, spleen, and lungs, or the activation of an immune response, which could lead to systemic inflammation and cell or tissue injury. Finally, the development of therapeutic nanotechnological devices must also take into account ethical and regulatory concerns associated with cutting-edge technologies; in particular, CRISPR-Cas9 is raising profound concerns regarding safety risks due to off-target mutations, possible eugenics through heritable germline editing, and potential worsening of socio-economic inequality [127]. This is the beginning of a great challenge, and it may be easily foreseen that, in the years to come, nanotechnological research will make strong efforts to provide effective tools to counteract sarcopenia, whose healthcare, social, and economic relevance is becoming higher and higher for the aging population in many countries [128].

## Figures and Tables

**Table 2:** Extracellular vesicles developed to counteract age-related muscle atrophy. The table summarizes the vesicle features, the experimental models where they were tested, the molecular factors or pathways involved in their action, and the main outcomes obtained.

Nanovector/Targeting Strategy	Cargo	Experimental Model	Factors/Pathways	Main Outcomes	Ref.
Adipose-derived EVs	microRNA	In vitro: C2C12 myoblasts/myotubes In vivo: mouse (18 months old); physiological aging model Route: intramuscular administration	ATG16L1, ATP, atrogin-1, BCL-2, BAX, caspase-3, caspase- 9, LC3A, LC3B MyHC, MYOD, myogenin, MuRF1, PAX7, p62, p-FOXO3/FOXO3, ROS, ULK1, ULK2	In vitro: reduced apoptosis and oxidative stress In vitro: attenuated mitochondrial autophagy, reduced ROS level and apoptosis	[97]
Myoblast-derived EVs modified with vesicular stomatitis virus glycoprotein	CRISPR/Cas9	In vitro: C2C12 myotubes In vivo: C57BL/6J mouse (8 weeks old); immobilization and denervation-induced muscle atrophy models Route: intramuscular administration	AKT, atrogin-1, FOXO3, mTOR, MuRF1, S6K, *miR-29b*	In vitro: maintenance of normal myotubes morphology, reduction in muscle atrophy markers In vivo: increased gastrocnemius muscle weight and cross-sectional area, activation and phosphorylation of anabolic factors, reduction in muscle atrophy markers	[98]
Plant-derived EVs	Bioactive component	In vitro: C2C12 myotubes In vivo: C57BL/6 mice (6 to 8 weeks old); dexamethasone-induced sarcopenic mouse model Route: oral administration	AKT/FOXO3/atrogin-1/MuRF1, AMPK/SIRT1/PGC-1α, beclin1, LC3B, MyHC, MYOD, myogenin, ROS, ATP, mitochondrial membrane potential	In vitro: restoration of cell morphology, inhibition of proteolysis and atrophy genes, stabilization of mitochondrial membrane potential, mitochondrial biogenesis In vivo: attenuation of muscle wasting, restoration of mitochondrial biogenesis and mitophagy, inhibition of proteolysis, modulation of gut-muscle axis	[99]
Myoblast-derived EVs enriched with HSP60	HSP60	In vitro: C2C12 myoblasts	PGC-1α	In vitro: upregulation of mitochondrial biogenesis, suppression of pathways triggering muscle protein degradation	[100]

Abbreviations: AKT, protein kinase B; AMPK, 5′ adenosine monophosphate-activated protein kinase; ATG16L1, autophagy related 16 like 1; ATP, adenosine triphosphate; BAX, BCL-2-associated X protein; BCL-2, B-cell lymphoma 2; CRISPR/Cas9, CRISPR-associated protein 9; EVs, extracellular vesicles; FOXO3, forkhead box O3; HSP60, heat shock protein 60; LC3A/3B, microtubule-associated protein 1 light chain 3A/3B; mTOR, mechanistic target of rapamycin; MuRF1: muscle RING finger 1; MyHC, myosin heavy chain; MYOD, myoblast determination protein 1; S6K, ribosomal protein S6 kinase beta-1; p-FOXO3, phosphorylated FOXO3; PAX7, paired Box 7; PGC-1α, peroxisome proliferator-activated receptor gamma coactivator-1α; ROS, reactive oxygen species; SIRT1, sirtuin 1; ULK1/2, unc-51 like autophagy activating kinase 1/2.

**Table 3:** Other organic nanovectors developed to counteract age-related muscle atrophy. The table summarizes the nanovector features, the experimental models where they were tested, the molecular factors or pathways involved in their action, and the main outcomes obtained.

Nanovector/Targeting Strategy	Cargo	Experimental Model	Factors/Pathways	Main Outcomes	Ref.
PEG-DSPE-based NPs functionalized with the muscle-homing modular peptide	Small-molecule inhibitor SW033291	In vitro: C2C12 myoblasts/myotubes In vivo: SAMP8 mouse; physiological aging model Route: intravenous administration	15-PGDH/prostaglandin E2	In vitro: biocompatibility of NPs In vivo: suppression of muscle wasting, preservation of mitochondrial morphology and function	[101]
Skeletal muscle cell membrane conjugated with muscle-homing peptide M12	Curcumin	In vitro: C2C12 myoblasts/myotubes In vivo: C57BL/6 mouse (8 week-old); D-galactose-induced aging model Route: intravenous injection	α-synuclein, GSH IL-6, IL-10, MDA, MYOD, myogenin, myostatin, p53, p38, SOD, SPHK1/SPNS2/S1PR, TNF-α	In vitro: prevention of cellular senescence, restoration of myogenic differentiation In vivo: reduced inflammation, enhanced motor capacity, increased skeletal muscle mass and myofiber size	[102]
Oil-in-water nanoemulsion	Astaxanthin	In vivo: C57BL/6J mouse (8 weeks old); dexamethasone-induced mouse model of muscle wasting Route: oral administration	4EBP1, atrogin-1, BNIP3, FOXO3, LC3B, MYOD, MuRF1, S6K, SDH, PGC-1α,	In vivo: preservation of skeletal muscle mass, preservation of structure, size, and alignment of myofibers	[103]
Hybrid PLGA-lipid NPs	PEA	In vitro: C2C12 myoblasts	/	In vitro: efficient internalization of NPs, improved cell viability	[104]

Abbreviations: BNIP3, BCL2 interacting protein 3; 4EBP1, eukaryotic translation initiation factor 4E-binding protein 1; FOXO3, forkhead box O3; GSH, glutathione; IL, interleukin; LC3B, microtubule-associated protein 1 light chain 3 beta; MDA, malondialdehyde; MuRF1, muscle RING finger 1; MYOD, myoblast determination protein 1; NPs, nanoparticles; S6K, ribosomal protein S6 kinase beta-1; PEA, palmitoylethanolamide; PEG-DSPE, polyethylene glycol-distearoylphosphatidylethanolamine; 15-PGDH, 15-hydroxyprostaglandin dehydrogenase; PGC-1α, peroxisome proliferator-activated receptor gamma coactivator-1α; PLGA, poly(lactic-co-glycolic acid); S1PR2, sphingosine-1-phosphate receptor 2; SAMP8, senescence-accelerated mice prone 8; SDH, succinate dehydrogenase; SOD, superoxide dismutase; SPHK1, sphingosine kinase 1; SPNS2, spinster homolog 2; TNF-α, tumor necrosis factor α.

**Table 4:** Inorganic nanoparticles developed to counteract age-related muscle atrophy. The table summarizes the nanoparticle features, the experimental models where they were tested, the molecular factors or pathways involved in their action, and the main outcomes obtained.

Nanovector/Targeting Strategy	Cargo	Experimental Model	Factors/Pathways	Main Outcomes	Ref.
Mesoporous silica NPs	Selenium and leucine	In vitro: C2C12 myotubes In vivo: C57BL/6 mouse; dexamethasone-induced sarcopenia mouse model and denervation-induced dystrophic sarcopenia mouse model Route: oral administration	AKT/FOXO3/MuRF1, DHE, GSH, GSH-PX4, mTOR/S6K, ROS	In vitro: reversed myotube atrophy, increased proliferation and differentiation, maintenance of redox homeostasis, preserved mitochondrial membrane and ultrastructure, inhibition of apoptosis In vivo: increased muscle mass and strength, restoration of redox homeostasis	[105]
Lipoic acid-modified gold NPs	Lipoic acid	In vitro: RAW 264.7 cells, C2C12 myoblasts/myotubes In vivo: C57BL/6J mouse (14–16 weeks old); glycerol-induced sarcopenia mouse model Route: local injection	Macrophage polarization	In vitro: shifting macrophages from pro-inflammatory (M1) to tissue-repairing (M2) phenotype In vivo: increased muscle mass and strength	[106]

Abbreviations: AKT, protein kinase B; DHE, dihydroethidium; FOXO3, forkhead box O3; GSH, glutathione; GSH-PX4, glutathione peroxidase 4; mTOR, mechanistic target of rapamycin; MuRF1, muscle RING finger protein-1; NPs, nanoparticles; ROS, reactive oxygen species; S6K, ribosomal protein S6 kinase.

**Table 5:** Nanoparticles developed as active agents to counteract age-related muscle atrophy. The table summarizes the nanoparticle features, the experimental models where they were tested, the molecular factors or pathways involved in their action, and the main outcomes obtained.

Nanovector/Targeting Strategy	Cargo	Experimental Model	Factors/Pathways	Main Outcomes	Ref.
Gouqi-derived nanovesicles	/	In vitro: C2C12 myotubes In vivo: C57BL/6J mouse (7 weeks old); dexamethasone-induced muscle atrophy Route: intramuscular administration	AMPK/SIRT1/PGC-1α, ATP, MYF5, MYOD, myogenin, OXPHOS	In vitro: increased myoblast proliferation, reduced myotube atrophy, restoration of mitochondrial function, upregulation of myogenic regulatory factors In vivo: prevention of muscle loss, increased myofiber cross-sectional area, enhanced strength, increased mitochondrial energy metabolism, activation of myogenic processes	[107]
Cerium oxide NPs	/	In vitro: C2C12 myoblasts In vivo: C57BL/6J mouse (52–54 week old); 4-hydroperoxy cyclophosphamide-induced sarcopenia Route: intramuscular administration	atrogin-1, CILP2, CXCL10, IL-6, ROS, serpinel/phospho-p21, TNF-α	In vitro: ROS scavenging, preservation of cellular morphology, downregulation of aging genes In vivo: decreased inflammation, prevention of muscle mass loss, increased muscle strength and myofiber cross-sectional area	[108]
Nanoscale colloidal dispersion	Kyungohkgo	In vitro: L6 myoblasts/myotubes In vivo: Sprague-Dawley rats (7 weeks old); dexamethasone-induced muscle atrophy Route: oral administration	atrogin-1, FOXO3, MyHC, MuRF1	In vitro: restoration of myotube size and structure In vivo: prevention of body weight loss, preservation of muscle mass and weight, and myofiber cross-sectional area, attenuation of muscle fibrosis	[109]

Abbreviations: AMPK, 5′ adenosine monophosphate-activated protein kinase; ATP, adenosine triphosphate; CILP2, cartilage intermediate layer protein 2; CXCL10, C-X-C motif chemokine ligand 10; FOXO3, forkhead box O3; IL-6, interleukin-6; MuRF1, muscle RING finger protein-1; MYF5, myogenic factor 5; MyHC, myosin heavy chain; MYOD, myoblast determination protein 1; NPs, nanoparticles; OXPHO, mitochondrial oxidative phosphorylation; PGC-1α, peroxisome proliferator-activated receptor gamma coactivator-1α; ROS, reactive oxygen species; SIRT1, sirtuin 1; TNF-α, tumor necrosis factor α.

**Table 6:** Nanomaterials developed as structural scaffolds to counteract age-related muscle atrophy. The table summarizes the nanomaterial features, the experimental models where they were tested, the molecular factors or pathways involved in their action, and the main outcomes obtained.

Nanomaterials	Cargo	Nanomaterials Properties	Experimental Model	Factors/Pathways	Main Outcomes	Ref.
Nanofibrillar-aligned collagen type I substrates	GFP+ primary mouse myogenic cells	Fiber alignment, topographical cues, porosity, stiffness	In vitro: primary mouse myoblasts In vivo: C57BL/6J mouse (8 and 80 weeks old); volumetric muscle loss defect Route: surgical implantation	CD31, CCL11, CXCL9, IL-6, IL-7, LIF	In vitro: cell differentiation and myotube formation In vivo: increased muscle mass strength and working performance, revascularization and innervation, immune modulation	[111]
Nanogratings	Extracellular vesicles	Topographical anisotropy (surface patterning, flat and nanografting)	In vitro: aged muscle stem/progenitor mouse cells In vivo: C57BL/6 mouse (3–6 and 21–25 months old); cardiotoxin-induced injury Route: intramuscular injection	Ki67, myogenin, MYOD, p38 MAPK	In vitro: increased proliferation, myotube formation, accelerated myogenic differentiation In vivo: increased myofiber cross-sectional area, activation of myogenic differentiation, attenuated fibrosis, recovery of muscle contraction function	[112]
Electrospun composite PCL, collagen I, and PEO nanofibers	/	Fiber alignment, topographical cues, porosity	In vitro: 3D co-culture system of human primary myoblasts and adipose-derived mesenchymal stromal cells	MyHC II, SMAD2/3	In vitro: enhanced cell alignment, upregulation of myogenic markers, increased fusion index	[113]
Electrospun PCL nanofibers functionalized with gold NPs and micropatterned with PEG hydrogel lines	/	Fiber alignment, topographical cues, micropatterning geometry, conductivity, stiffness	In vitro: C2C12 myoblasts/myotubes	IGF-1 signaling pathway, myogenin, MYOD, myostatin	In vitro: enhanced cell alignment, differentiation and fusion, upregulation of pro-myogenic signaling pathways	[114]

Abbreviations: CCL11, C-C motif chemokine ligand 11; CD31, cluster of differentiation 31; CXCL9, C-X-C motif chemokine ligand 8; GFP, green fluorescent protein; IGF-1, insulin-like growth factor-1; IL, interleukin; LIF, leukemia inhibitory factor; MyHC II, myosin heavy chain II; MYOD, myoblast determination protein 1; NPs, nanoparticles; p38 MAPK, p38 mitogen-activated protein kinase; PCL, poly-ε- caprolactone; PEG, polyethylene glycol; PEO, polyethylene oxide; SMAD2/3, small mother against decapentaplegic homolog 2 and 3.

**Table 7:** Nanomaterials with dual function developed to counteract age-related muscle atrophy. The table summarizes the nanomaterial features, the experimental models where they were tested, the molecular factors or pathways involved in their action, and the main outcomes obtained.

Nanomaterials	Cargo	Nanosystems Characteristics	Experimental Model	Factors/Pathways	Main Outcomes	Ref.
Thermosensitive polaxamer hydrogel	CD146, IGF-1, collagen I and III.	Multifunctionality, stimuli responsiveness, immunomodulation	In vitro: C2C12 myoblasts/myotubes In vivo: mouse (3 and 24 months old); BALB/c-nu/nu and lipopolysaccharide-induced muscle injury mouse models Route: subcutaneous injection	AREG, ATG5, ATG7, beclin-1, BNIP3, CD11b, CD206, CD146/VEGFR-2, eMyHC, LC3B-II, myogenin, MyHC II, MYOD, p62	In vitro: promotion of cells viability, differentiation and migration, suppression of apoptosis, increased efferocytosis and phagocytic activity In vivo: increased muscle mass, upregulation of myogenic regulatory factors, activation of autophagy, suppression of atrophy and inflammation	[115]
Hyaluronic acid hydrogel	Exosomes	Multifunctionality, immunomodulation	In vitro: Sprague-Dawley rats (15 months old); physiological aging model with an induced chronic rotator cuff tear injury Route: local administration	ARG1, CD206, BMP4/SMAD1/5/9, COL1A1, COL2A1, IL-4, IL-10, NF-κB, OCN, p16, p21, RUNX2, SCX, SOX-9	In vivo: anti-inflammatory shift, attenuated cellular senescence, improved bone mineral density and volume, promotion of regenerative processes at the tendon-to-bone interface	[116]
Diamido-PEGylated black phosphorus nanosheets functionalized with mitochondrial-derived peptide MOTS-c	MOTS-c	Multifunctionality, immunomodulation	In vitro: C2C12 myoblasts/myotubes In vivo: C57BL/6J mouse (4 and 20 months old); physiological aging model Route: intravenous injection	ATP, γ-H2AX, MAPK, PI3K/AKT/NRF2, RNS, ROS/p38, SIRT1, SIRT3, TOM20, VDAC-1	In vitro: attenuated cellular senescence and DNA damage, normalized mitochondrial function, reduced oxidative stress In vivo: increased muscle mass, improved muscle function, normalized mitochondrial function, anti-inflammatory effects	[117]
Ultrashort peptide-based hydrogel	/	Immunomodulation	In vitro: C2C12 myoblasts/myotubes In vivo: C57BL/6 mouse; dexamethasone-induced sarcopenia Route: intramuscular/local administration	IGF-1 receptor, myogening, MyHC, MYOD, PI3K/AKT/insulin	In vitro: enhanced myoblasts proliferation and differentiation, upregulation of myogenesis factors, reduced apoptosis In vivo: increased muscle mass, prevention of muscle tissue fibrosis	[118]
Two-dimensional layered double hydroxide	Urolithin A, metal ions	Multifunctionality, immunomodulation	In vitro: C2C12 myoblasts/myotubes, primary mouse muscle satellite cells, RAW 264.7 cells, HUVEC In vivo; rat (18–20 months old); dexamethasone-induced rat model of sarcopenia Route: intravenous injection	AMPK/SIRT1/PGC-1α, BCL-xL/BIM, cyclin D1, GLUL, HIF-1α, IGF-1, IL-10, myogenin, MYF5, MyHC-β, MYOD, MuRF1, SOD, VEGF	In vitro: mitigated cell atrophy, enhanced differentiation and fusion, upregulated myogenesis factors, restored mitochondrial function, macrophage polarization, angiogenic effects In vivo: increased muscle mass, enhanced grip strength, promotion of vasculogenesis, restored mitochondrial function	[119]
Liposomes in energy-replenishing HAMA microspheres	NMN	Multifunctionality	In vitro: C2C12 myoblasts/myotubes In vivo: C57BL/6 J mouse (10 weeks old); dexamethasone-induced sarcopenia/muscle wasting model Route: local injection	AMPK/SIRT1/PGC-1α, ATP, atrogin-1, COXIV, desmin, MyHC, MYOD, MuRF1, NAD+/NADH, p16, p53, p21, ROS	In vitro: restored mitochondrial function, inhibition of cellular senescence, prevention of cell atrophy In vivo: increased muscle mass, strength and motor function, restored mitochondrial function	[120]
Thiol-rich human serum albumin NPs	Edaravone	Multifunctionality, stimuli responsiveness	In vitro: C2C12 myoblasts, A549 cells, HepG2 cells In vivo: ICR mouse (4 weeks old); a disuse muscle atrophy model induced by a 14-day hindlimb unloading (tail-suspension procedure) Route: intravenous administration	atrogin-1, CPK, LPO, MDA, myostatin, PGC-1α, SPARC	In vitro: mitigated oxidative stress, downregulation of muscle-wasting factors, preservation of mitochondrial structure and function In vivo: prevention of muscle mass loss, improved muscle strength, reduced muscle injury markers and oxidative stress	[121]
Dual-targeted hybrid nanovesicles engineered by fusing iMSC-EVs with peptide-modified liposomes	*miR-206-5p*	Multifunctionality, dual targeting, immunomodulation	In vitro: bone marrow mesenchymal stem cells, mouse muscle satellite cells In vivo: KM mouse (adult); murine model of osteosarcopenia Route: intravenous administration	DUSP4/p38, IL-6, MAPK, MyHC, MYOD, myogenin, OCN, OPN, OSX, RUNX2	In vitro: reversed cellular senescence, restored mitochondrial function, enhanced myogenesis, accelerated osteogenesis, stimulation of protective immune response In vivo: musculoskeletal structural preservation, restored muscle mass and strength	[122]
Lead-free (K, Na) NbO_3_ NPs in a piezoelectric hydrogel	/	Multifunctionality, stimuli responsiveness, immunomodulation	In vitro: C2C12 myoblasts/myotubes In vivo: C57BL/6 mouse (9 to 10 weeks old); volumetric muscle loss model Route: local injection	Calcium-NFAT, IL-17, iNOS, MEF2C, MYF5, MyHC, myogenin, NF-кB, PGP9.5, TNF-α	In vitro: accelerated myoblasts migration, promotion of myogenic differentiation, activated calcium influx, acceleration of macrophage-associated inflammation resolution In vivo: recovery of muscle mass and strength, enhanced nerve repair, reduced inflammation, establishment of pro-regenerative microenvironment	[123]

Abbreviations: AKT, protein kinase B; AMPK, 5′ adenosine monophosphate-activated protein kinase; AREG, amphiregulin; ARG1, arginase-1; ATG5 or 7, autophagy-related 5 or 7; ATP, adenosine triphosphate; BCL-xL, B-cell lymphoma-extra large; BIM, BCL-2 interacting mediator of cell death; BNIP3, BCL2/Adenovirus E1B 19 kDa Protein-Interacting Protein 3; BMP4, bone morphogenetic protein 4; CD, cluster of differentiation; COL1A1, collagen type I alpha 1 chain; COL2A1, collagen type II alpha 1 chain; COXIV, cytochrome c oxidase subunit 4; CPK, plasma creatine phosphokinase; DNA, deoxyribonucleic acid; DUSP4, dual specificity phosphatase 4; eMyHC, embryonic myosin heavy chain; γ-H2AX, gamma-H2AX/phosphorylated histone H2AX; GLUL, glutamate-ammonia ligase; HAMA, hyaluronic acid methacrylate; HIF-1α, hypoxia-inducible factor 1α; IGF-1, insulin-like growth factor 1; IL, interleukin; iMSC-EVs, induced pluripotent stem cells-derived mesenchymal stem cell extracellular vesicles; iNOS, inducible nitric oxide synthase; LC3B-II, microtubule-associated protein 1 light chain 3B; LPO, lipid peroxidation; MAPK, mitogen-activated protein kinase; MDA, malondialdehyde; MEF2C, myocyte-specific enhancer factor 2C; MyHC: myosin heavy chain; MOTS-c, mitochondrial open reading frame of the 12S rRNA type-c; MuRF1, muscle RING finger 1; MYF5, Myogenic Factor 5; MYOD, myoblast determination protein 1; NAD+, nicotinamide adenine dinucleotide; NADH, nicotinamide adenine dinucleotide reduced form; NF-κB, nuclear factor kappa-light-chain-enhancer of activated B cells; NFAT, nuclear factor of activated T-cells; NMN, nicotinamide mononucleotide; NPs, nanoparticles; NRF2, nuclear factor erythroid 2-related factor 2; OCN, osteocalcin; OPN, osteopontin; OSX, osterix; PEG, polyethylene glycol; PGC-1α, peroxisome proliferator-activated receptor gamma coactivator-1α; PGP9.5, protein gene product 9.5; PI3K, phosphatidylinositol 3-kinase; RNS, reactive nitrogen species; ROS, reactive oxygen species; RUNX2, runt-related transcription factor 2; SCX, scleraxis; SIRT, sirtuin; SMAD1/5/9, small mother against decapentaplegic homolog 1, 5, and 9; SOD, superoxide dismutase; Sox-9, SRY-Box transcription factor 9; SPARC, secreted protein acidic and rich in cysteine; TNF-α, tumor necrosis factor α; TOM20, translocase of outer mitochondrial membrane 20; VDAC-1, voltage-dependent anion channel 1; VEGF, vascular endothelial growth Factor; VEGFR-2, vascular endothelial growth factor receptor-2.

## Data Availability

No new data were created or analyzed in this study. Data sharing is not applicable to this article.
